# Frontal, Parietal, and Temporal Brain Areas Are Differentially Activated When Disambiguating Potential Objects of Joint Attention

**DOI:** 10.1523/ENEURO.0437-19.2020

**Published:** 2020-10-19

**Authors:** P.M. Kraemer, M. Görner, H. Ramezanpour, P.W. Dicke, P. Thier

**Affiliations:** 1 Department of Cognitive Neurology, Hertie Institute for Clinical Brain Research, University of Tübingen, Tübingen 72076, Germany; 2 Graduate School of Neural and Behavioural Sciences, University of Tübingen, Tübingen 72074, Germany; 3 International Max Planck Research School for Cognitive and Systems Neuroscience, University of Tübingen, Tübingen 72074, Germany; 4 Werner Reichardt Centre for Integrative Neuroscience, University of Tübingen, Tübingen 72076, Germany; 5 Department of Psychology, University of Basel, Basel 4055, Switzerland

**Keywords:** fMRI, gaze following, human lateral intraparietal area, inferior frontal junction, joint attention, superior temporal sulcus

## Abstract

Humans establish joint attention with others by following the other’s gaze. Previous work has suggested that a cortical patch (gaze-following patch, GFP) close to the posterior superior temporal sulcus (pSTS) may serve as a link between the extraction of the other’s gaze direction and the resulting shifts of attention, mediated by human lateral intraparietal area (hLIP). However, it is not clear how the brain copes with situations in which information on gaze direction alone is insufficient to identify the target object because more than one may lie along the gaze vector. In this fMRI study, we tested human subjects on a paradigm that allowed the identification of a target object based on the integration of the other’s gaze direction and information provided by an auditory cue on the relevant object category. Whereas the GFP activity turned out to be fully determined by the use of gaze direction, activity in hLIP reflected the total information needed to pinpoint the target. Moreover, in an exploratory analysis, we found that a region in the inferior frontal junction (IFJ) was sensitive to the total information on the target. An examination of the BOLD time courses in the three identified areas suggests functionally complementary roles. Although the GFP seems to primarily process directional information stemming from the other’s gaze, the IFJ may help to analyze the scene when gaze direction and auditory information are not sufficient to pinpoint the target. Finally, hLIP integrates both streams of information to shift attention to distinct spatial locations.

## Significance Statement

Our paper captures work deploying fMRI to identify brain structures and mechanisms that allow us to pinpoint the object attended by the other out of the many hit by her/his gaze vector. Our results suggest that an area at the border between premotor and prefrontal cortex plays a major role in providing the complementary information needed by the temporo-parietal underpinnings of gaze following to shift the observer’s attention to the correct object. Our results support the interplay of a network of distributed elements with distinct functional contributions allowing us to deploy Joint Attention, a key underpinning of viable social behavior and a full-fledged theory of (the other’s) mind (TOM).

## Introduction

We follow the other’s gaze to objects of her/his attention which allows us to shift our own attention to the same object and to thereby establish joint attention. By associating our object-related intentions, expectations and desires with the other one, joint attention allows us to develop a theory of (the other’s) mind (TOM; Emery, 2000). TOM is a major basis of successful social interactions (Baron-Cohen, 1994, 1995), and arguably, its absence is at the core of neuropsychiatric disorders such as autism.

Human gaze following is geometric (Butterworth and Jarrett, 1991; Atabaki et al., 2015). This means that we use the other’s gaze vector to identify the exact location of the object of interest. The features of the human eye such as the high contrast between the white sclera and dark iris allow us to determine the other’s eye direction at high resolution (Kobayashi and Kohshima, 1997; Bock et al., 2008). However, knowledge of direction is not sufficient to pinpoint an object in 3D. In principle, differences between the directions of the two eyes, i.e., knowledge of the vergence angle, could be exploited to this end. Yet, this will work only for objects close to the beholder as the angle will become imperceptibly small if the objects are outside the confines of peripersonal space. On the other hand, gaze following remains precise also for objects quite far from the other one although the gaze vector will in many cases hit more than one object (Butterworth and Jarrett, 1991). Hence, how can these objects be disambiguated? We hypothesized that singling out the relevant object is a consequence of recourse to prior information on the objects and their potential value for the other. For instance, let us assume that the day is hot and that the other’s appearance may suggest thirst and the desire to take a sip of something cool. If her/his gaze hits a cool beverage within a set of other objects of little relevance for a thirsty person, the observer might safely infer that the beverage is the object of desire. In this example, gaze following is dependent on prior assumptions about the value of objects for the other. Of course, the value the object may have for the observer also matters. For instance, Liuzza et al. (2011) showed that an observer’s appetence to follow the other’s gaze to portraits of political leaders is modulated by the degree of political closeness. If the politician attended by the other was a political opponent of the observer, the willingness to follow gaze was significantly reduced. Also knowing that gaze following may be inadequate in a given situation and that the other may become aware of an inadequate behavior will suppress it (Teufel et al., 2009, 2010). However, only assumptions about the object value of the other will help to disambiguate the scene.

Following the gaze of others to a particular object is accompanied by a selective BOLD signal in an island of cortex in the posterior superior temporal sulcus (pSTS), the gaze-following patch (GFP; Materna et al., 2008; Laube et al., 2011; Marquardt et al., 2017). In these studies, the target object could be identified unambiguously by gaze direction as for a given gaze direction the vector hit one object only. Hence, it remains unclear whether the GFP helps to integrate the information needed to disambiguate the object choice in case the gaze vector hits more than one object. In order to address this question, we conducted an fMRI study in which the selection of the object of joint attention required that the observer resorted to another source of information aside from the gaze cue.

## Materials and Methods

### Participants

Nineteen healthy, right-handed volunteers (nine females and 10 males, mean age 27.4, SD = 3.6) participated in the study over three sessions. Participants gave written consent to the procedures of the experiment. The study was approved by the local Ethics Review Board and was conducted in accordance with the principles of human research ethics of the Declaration of Helsinki.

### Task and procedure

The study was conducted in three sessions across separate days. On day 1, we instructed participants about the study goals and familiarized them with the experimental paradigms outside the MRI-scanner by carrying out all relevant parts of the fMRI experiments. The following fMRI experiments included a functional localizer paradigm for the scanning session on day 2 as well as a contextual gaze following paradigm for the scanning session on day 3.

#### Behavioral session

After participants had been familiarized with the tasks, they were head-fixed using a chinrest and a strap to fix the forehead to the rest. Subjects were facing toward a frontoparallel screen (resolution = 1280 × 1024 pixels, 60 Hz; distance to eyes ≈ 600 mm). Eye tracking data were recorded while participants had to complete 80 trials of the localizer paradigm and 72 trials of contextual gaze following.

#### Localizer task

We resorted to the same paradigm used in (Marquardt et al., 2017) to localize the gaze following network and in particular its core, the GFP. In this paradigm, subjects were asked to make saccades to distinct spatial targets based on information provided by a human portrait presented to the observer. Depending on the instruction, subjects either had to rely on the seen gaze direction to identify the correct target (gaze following condition) or, alternatively, they had to use the color of the irises, changing from trial to trial but always mapping to one of the targets, to make a saccade to the target having the same color (color mapping condition). In other words, the only difference between the two tasks was the information subjects had to exploit to solve the task, while the visual stimuli were the same.

This task is associated with higher BOLD activity in the GFP, a region close to the posterior end of the STS, when subjects performed gaze following compared with color mapping. The task is further associated with the activation of regions in the posterior parietal cortex as well as the frontal cortex that take part in controlling spatial attention and saccade generation (Materna et al., 2008; Marquardt et al., 2017). Out of the 19 subjects of our study, 16 performed six runs (40 trials per run) and for reasons of time management during image acquisition, one subject performed five runs and two subjects performed four runs.

#### Contextual gaze following task

**Figure 1.**
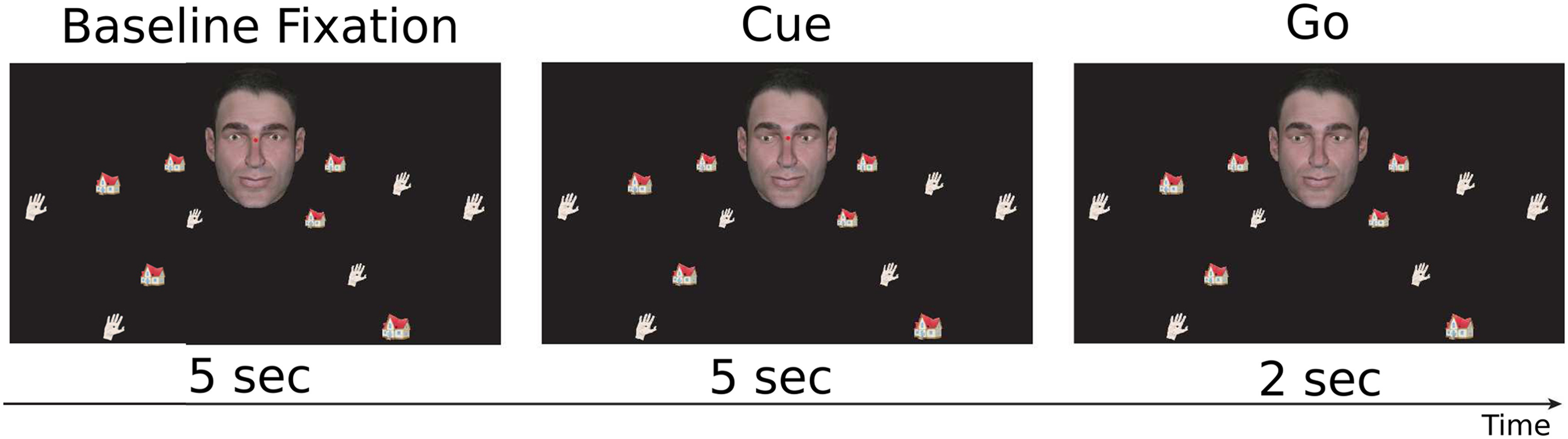
Contextual gaze following task. An avatar appeared in the center of the screen together with four linearly arranged sets of objects (houses and hands). After a baseline fixation period, the portrait’s gaze shifted toward one specific target object simultaneously with an auditory contextual instruction specifying the object class of the target (“hand” or “house”) or not, i.e., remaining uninformative (“none”). While maintaining fixation, subjects needed to decide on the target and make a saccade to the chosen target after a go-signal which was indicated by the disappearance of the fixation dot.

An example of a trial is shown in Figure 1. Each trial consisted of the following sequence of events. The trial started with the appearance of the portrait of an avatar (6.7° × 10.5° of visual angle) image in the center of the screen together with four arrays of drawn objects (houses and hands, three objects per array). Subjects were asked to fixate on a red fixation dot between the avatar’s eyes. After 5 s of baseline fixation, the avatar’s eye gaze shifted toward one specific target object. Simultaneously, a spoken instruction either specified the object class of the target (spoken words “hand” or “house”) or was not informative (“none”). While maintaining fixation, subjects needed to judge which object the avatar was looking at. After 5-s delay, the fixation dot vanished, an event that served as the go-signal. Participants had 2 s to make a saccade to the chosen target object and fixate it until a subsequent blank fixation screen was presented for ∼8 s. The subjects were instructed to perform the task as accurately as possible. They were specifically instructed, when unsure about the actual target, nevertheless to rely on gaze and contextual information and choose the target they believed the avatar to be looking at.

The information provided by the spoken instruction distinguished three experimental conditions, one unambiguous, and two ambiguous conditions: ambiguous-informative and ambiguous-uninformative. The verbal instruction in the unambiguous condition reduced the number of potential targets from three to one by naming the object category with only one representative in the array. For instance, in Figure 1, the avatar gazes at the lower left array, specifying two hands and one house as potential gaze targets. An unambiguous instruction would be the auditory cue “house.” The ambiguous-informative instruction in this example, “hand,” reduced the number of potential gaze targets to two. In the ambiguous-uninformative condition, the instruction would have been “none,” not suited to reduce the number of potentials targets.

Participants performed six blocks of 30 trials each (10 per condition), summing up to 180 trials in total.

### Stimuli

Visual and auditory stimuli as well as data collection was controlled by a custom-made Linux-based system. The stimuli in the localizer task were designed according to the stimuli used in a previous study (Marquardt et al., 2017). The stimuli of the contextual gaze following task consisted of an avatar and in total 12 target objects from two categories (houses and hands). The avatar was generated with a custom-made OpenGL library which offers a controlled virtual 3D environment in which an avatar can be set to precisely gaze at specific objects. More specifically, the program allows objects to be placed on a circle, parallel to the coronal axis, and anterior to the avatar face. For each stimulus, we placed 12 objects in the surroundings of the avatar. The location of individual objects was fully determined by the distance to the coronal plane at the level of the avatar’s nasion, the radius of the circle and the angle of the object on that circle. By keeping the angle on the circle constant for each set of three objects, we created four arrays at angles 120°, 150°, 210°, and 240°. The individual locations of these objects were specified by varying the distance and the circle radii based on trigonometric calculations. For these calculations we assumed a right triangle from the avatar’s nasion with the hypotenuse pointing toward the object, an adjacent leg (length corresponded to the distance of the circle) proceeding orthogonal to the coronal plane, and an opposite leg which corresponded to the radius. By keeping tanα fixed to 0.268, we varied the distances and circle radii. For the 120° and 240° arrays, the circle radii were 335, 480, and 580 and the distances were 90, 129, and 151 virtual mm. For the 150° and 210° arrays, the radii were 380, 510, and 590 and the distances were 102, 137, and 158 virtual mm. The reason for the difference of radii and distances between 120°/240° and 150°/210° arrays was that this allowed the total width of the screen to be exploited. This procedure guaranteed that the angle of the gaze vector to all objects on the array was almost identical. This makes it relevant to take contextual information into account to choose the true target.

The objects were drawings of the two categories houses and hands, downloaded from freely available online sources (https://all-free-download.com/free-vector/download/5-house-vector_154512.html, https://www.freepik.com/free-vector/hand-drawn-hands_812824.htm#term=hands&page=1&%20position=37). The target objects were arranged in four radial directions (three objects in each direction) with the avatar’s eyes as the origin; in other words, the avatar’s gaze always hit one out of three objects along the gaze vector though participants were not able to tell which of the three it was. On each array, either two hands and one house or one hand and two houses were present. Further, we fixed the number of hands and houses per hemifield to three. The relative order of the objects was pseudo-randomized from trial to trial.

We created a pool of stimulus sets which satisfied three constraints: There was an equal number of trials in which (1) the targets were hands or houses, (2) targets were presented with an unambiguous, ambiguous-informative and ambiguous-uninformative instruction, and (3) the spatial position (one out of twelve potential positions) of targets was matched. This led to 72 stimulus sets. We exposed every subject to 180 trials in which each stimulus set was shown twice and for the residual 36 trials, stimuli were drawn pseudo-randomly from the stimulus pool so that the three aforementioned criteria were met.

Auditory instructions were delivered via headphones (Sennheiser HD 201 during the behavioral session, and standard air pressure headphones of the scanner system during the MRI sessions). The auditory instructions “hand,” “house,” and “none” were computer generated with the web application imTranslator (http://imtranslator.net/translate-and-speak/speak/english/) and processed with the software Audacity 2.1.2. The sound files had a duration of 600 ms.

### Eye tracking

During all three sessions, we recorded eye movements of the right eyes using commercial eye tracking systems (behavioral sessions: Chronos Vision C-ETD, sampling rate 400 Hz, resolution < 1° visual angle; scanning sessions: SMI iView X MRI-LR, sampling rate = 50 Hz, resolution ≈ 1° visual angle).

Eye tracking data were processed as follows. First, we normalized the raw eye tracking signal by dividing it by the average of the time series. Eye blinks were removed using a velocity threshold (>1000°/s visual angle). Next, we focused on a time window in which we expected the saccades to the target objects to occur ([go-signal – 500 ms, go-signal + 1800 ms]). Within this time window, we detected saccades by identifying the time point of maximal eye movement velocity. Presaccadic and postsaccadic fixation positions were determined by averaging periods of 200 ms before and after the saccade occurred. Due partly to extensive noise of the eye tracking signal recorded in the scanner, we did not automatize the categorization of the final gaze position. Instead, we plotted *x*- and *y*-coordinates of the postsaccadic eye position for every run that was not contaminated by too much noise. An investigator (M.G.), who was blind to the true gaze target directions of the stimulus face, manually validated which trials yielded positions that were clearly assignable to a distinct object location. For the behavioral analysis we only used valid trials (mean number of valid trials per participant = 80.2, SD =* *45.4, range = [0,153]) and weighted the individual performance values by its number to compute weighted means and SDs. Note that we used these valid trials only for the behavioral analysis but used all trials of the participants for the fMRI analysis, assuming that eye tracking measurement noise was independent of the performance of the subjects.

### fMRI acquisition and preprocessing

We acquired MR images using a 3T scanner (Siemens Magnetom Prisma) with a 20-channel phased array head coil. The head of the subjects was fixed inside the head coil by using plastic foam cushions to avoid head movements. An AutoAlign sequence was used to standardize the alignment of images across sessions and subjects. A high-resolution T1-weighted anatomic scan (MP-RAGE, 176 × 256 × 256 voxel, voxel size 1 × 1×1 mm) and local field maps were acquired. Functional scans were conducted using a T2^*^-weighted echo-planar multibanded 2D sequence (multiband factor = 2, TE = 35 ms, TR = 1500 ms, flip angle = 70°) which covered the whole brain (44 × 64 × 64 voxel, voxel size 3 × 3 × 3 mm, interleaved slice acquisition, no gap).

For image preprocessing we used the MATLAB SPM12 toolbox (Statistical Parametric Mapping, https://www.fil.ion.ucl.ac.uk/spm/). The anatomical images were segmented and realigned to the SPM T1 template in Montreal Neurological Institute (MNI) space. The functional images were realigned to the first image of each respective run, slice-time corrected and coregistered to the anatomical image. Structural and functional images were spatially normalized to MNI space. Finally, functional images were spatially smoothed with a Gaussian kernel (6 mm full-width at half maximum).

### fMRI analysis

We estimated a generalized linear model (GLM) to identify regions of interest (ROIs) of single subjects. On these regions, we performed time course analyses to investigate event-related BOLD signal changes. In a first-level analysis, we estimated GLMs for the localizer task (GLM_loc_) and the contextual gaze following task (GLM_cgf_). The GLM_loc_ included predictors for the onset of directional cues and of the baseline fixation phase. The GLM_cgf_ had predictors for the onset of the contextual instruction coinciding with the gaze cue. These event-specific predictors of the two GLMs used the canonical hemodynamic response function of SPM to model the data. We corrected for head motion artifacts by the estimation of six movement parameters with the data of the realignment preprocessing step. Low-frequency drifts were filtered using a high-pass filter (cutoff at 1/128 Hz).

#### GFP and hLIP localizer

Before collecting the data, we specified the expected locations of two brain areas from the literature. We resorted to the parietal coordinates of the human homolog of the monkey area LIP (hLIP) which had been identified using a delayed saccade task (Sereno et al., 2001). The GFP standard coordinates were taken from Marquardt et al. (2017). We transformed the standard coordinates for the hLIP and the GFP from Talairach space into MNI space, using an online transformation method of Lacadie and colleagues (Lacadie et al., 2008; http://sprout022.sprout.yale.edu/mni2tal/mni2tal.html).

To identify ROIs at the group level, we compared β weights of the statistical parametric maps from the GLM_loc_ in a second-level analysis. The GFP weights were derived from the contrast gaze following versus color mapping, and the hLIP weights from the contrast gaze following versus baseline fixation. To be characterized as GFP or hLIP, a cluster’s maximum weight had to be located in close proximity to their respective standard coordinates.

We aimed to identify ROIs on an individual subject level. To this end, we used the contrast maps from the first-level analysis of the GLM_loc_. We selected the coordinates of the maximum contrast voxel which minimized the distance to the group level coordinates. This voxel had to be part of a statistically significant cluster (cluster size ≥ 6, *p *<* *0.05). Because of relatively low signal-to-noise ratio in the gaze following versus color mapping contrast, and the corresponding increased risk of false-positive activations, we decided to introduce a second criterion to make GFP localization more rigorous in single subjects. This proximity criterion additionally required the cluster to be located at least partially within 10 mm from the group level coordinates of the respective ROI.

##### Contrasts of context conditions

In addition to our a priori ROIs, we were interested in whether the contextual gaze following task might also activate regions which we did not consider beforehand. We performed a whole-brain analysis on the data from the contextual gaze following task. Using the GLM_cgf_, we contrasted the weights of the two ambiguous conditions with the unambiguous condition at the group level (second-level analysis, significance threshold *p *<* *0.001, cluster size ≥ 6 voxel) as well as at the single subject level (first-level analysis, significance threshold *p *<* *0.05, cluster size ≥ 6 voxel).

#### Time course analysis

We determined the individual time courses of the BOLD signal within sphere-shaped ROIs. Whenever we identified an ROI on the single-subject level, spheres with a radius of 5 mm were centered at the individual ROI coordinates. In case the identification of a ROI on the single-subject level was not possible, we deployed spheres with a radius of 10 mm centered at the group level location, assuming these spheres would capture relevant single-subject activity.

For every subject and sphere, raw time series of the BOLD signal were extracted using the MATLAB toolbox *marsbar 0.44* (http://marsbar.sourceforge.net). Because of technical problems in the reconstruction of trial times, for five participants we included only five runs and for two only four runs into the analysis. The time course of every trial was normalized by the average signal intensity 5 s before the onset of the contextual instruction and transformed into % of signal change. For each participant, we averaged time courses across trials and runs and used the time courses of the three contextual conditions in the six ROIs for our analysis. To test differences across conditions for statistical significance, we performed permutation tests at each time point after contextual instruction delivery. To do so, we pooled the data of two experimental conditions, respectively, and produced 10,000 random splits for each pool. By computing the differences between the means of these splits, we obtained a distribution of differences under the null hypothesis. Calculating the fraction of values more extreme than the actual difference between means allowed us to obtain a *p* value for each time bin. To account for the multiple comparison problem, we transformed *p* values into FDR corrected *q* values (Benjamini and Hochberg, 1995) and considered each time bin with *q *<* *0.05 as statistically significant.

We conducted two additional analyses, the first one to obtain Bayesian credible intervals (BCIs) for the time courses and a second one, a “searchlight” analysis which scanned the whole brain for voxels whose signal could be used to decode the unambiguous versus the ambiguous-uninformative conditions.

For the first we estimated hierarchical models for each experimental condition and ROI allowing the intercept to vary for each participant. The models were linear combinations of 7 sinusoidal basis functions. Model estimation was conducted using the *nideconv* package (de Hollander and Knapen, 2017) which interfaces with the Stan probabilistic programming language for Bayesian model estimation (Stan Development Team, 2018); 95% BCI indicate that, given the data and the model, the data generating parameter is included in the interval with a probability of 95%.

For the second we used the β-images from the first-level analysis as input to a searchlight algorithm that estimated classification accuracies for each voxel. We employed the TDT toolbox (Hebart et al., 2015) and trained SVMs (leave-one-run-out cross-validated) to classify unambiguous and ambiguous-uninformative conditions. This yielded one map per participant representing the estimated classification accuracies minus the chance-level of 0.5. For a subsequent second-level analysis these individual maps were then smoothed (4-mm kernel) and fed into SPM’s second-level pipeline to obtain a group level t-map as suggested by Hebart et al. (2015). Only voxels with a value corresponding to a *p* value smaller or equal to 0.001 were included into the end result. Since classification accuracies do not follow a normal distribution computing a t-map is not the ideal method as it is likely to provide an overestimation of significance. However, even if an approach tending to overestimate statistical significances fails to detect areas of significant classification, this method can still safely be considered conservative. This was the case with respect to GFP, whose possible involvement in the differentiation of the various conditions we wanted to reexamine using the searchlight analysis. As a final step we used the same ROIs that were used in the time course analysis to extract the local accuracies (minus chance-level) from each participant’s accuracy map. The obtained distributions across participants were tested against the result of an ideal ignorant classifier that always performs at chance level using a Wilcoxon signed-rank test.

### Code accessibility

All data and analysis scripts are freely available on https://figshare.com/projects/Contextual_Gaze_Following/78222.

## Results

Our subjects participated in two fMRI experiments. The first one was a localizer task that allowed us to identify two ROIs of which we know are relevant for attentional shifts based on social cues, the GFP and hLIP (Materna et al., 2008; Marquardt et al., 2017). Our main intention was to investigate the BOLD activity of these regions in a contextual gaze following task (experiment 2). In this task, the subjects used the gaze direction of a human avatar, complemented by a spoken instruction. In one out of three conditions the observer was able to unambiguously identify the relevant object out of several hit by the other’s gaze vector. This was the case in the unambiguous condition in which the spoken instruction identified an object class represented by only one exemplar on the avatar’s gaze vector. In the two other conditions (to which we refer collectively as ambiguous conditions), the spoken information was insufficient either because two exemplars of the relevant object category were available (ambiguous-informative condition) or because the verbal instruction was uninformative (ambiguous-uninformative condition). In the latter case, observers were left with a choice between three objects.

### Behavioral performance

**Figure 2.**
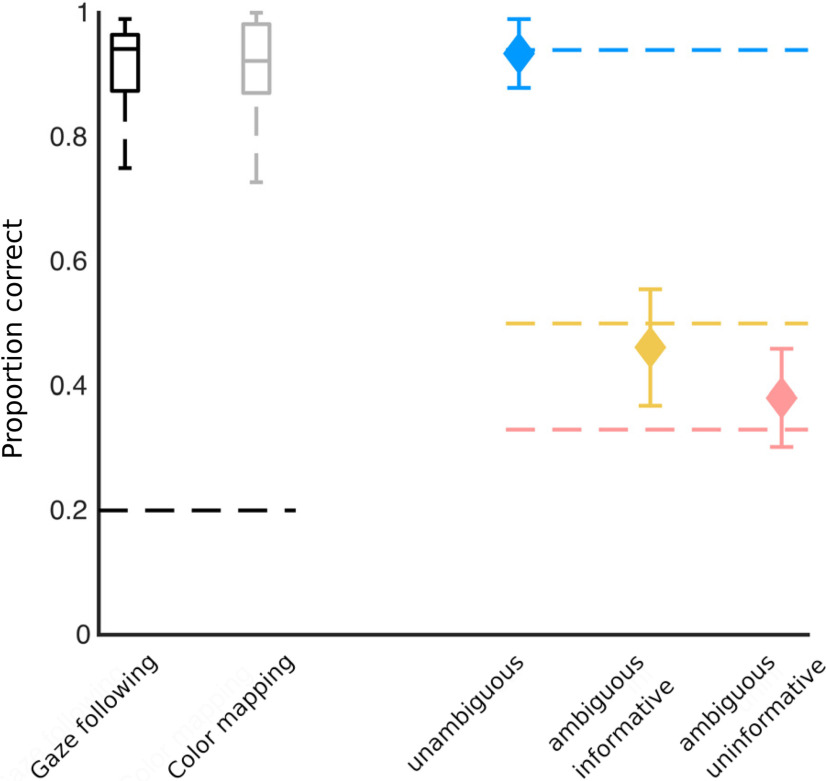
Behavioral performance. Left, Boxplots (black and gray) showing the percentage of correct response in the localizer paradigm (dashed line depicts chance level performance, see Extended Data Fig. 2-1 for a description of the localizer paradigm). Right, Plots of correct responses in the contextual gaze following paradigm (weighted mean performance and weighted SD, dashed lines depict expected performance; blue: unambiguous, yellow: ambiguous-informative, salmon: ambiguous-uninformative).

In the localizer task, subjects were able to hit targets reliably and without significant difference between the two conditions (median hit rates: gaze following: 0.94 ± 0.13 SD; color matching: 0.92 ± 0.09 SD; *p *=* *0.6, two-tailed *t* test, *N *=* *19; Fig. 2). Using the gaze following performance in the localizer task as reference we assumed the following expected hit rates for the contextual gaze following task: 0.94 for the unambiguous condition, 0.94*1/2 for the ambiguous-informative, and 0.94*1/3 for the ambiguous-uninformative condition (Fig. 2). As summarized in Figure 2, the measured performances matched the assumptions in the contextual gaze following task very well (comparison by two-tailed *t* tests, n.s.). This result indicates that the probability of identifying an object as a target was determined by the information provided by gaze direction and the verbal instruction.

10.1523/ENEURO.0437-19.2020.f2-1 Extended Data Figure 2-1 Description of the localizer paradigm. Download Figure 2-1, DOC file.

### ROI localization

**Figure 3.**
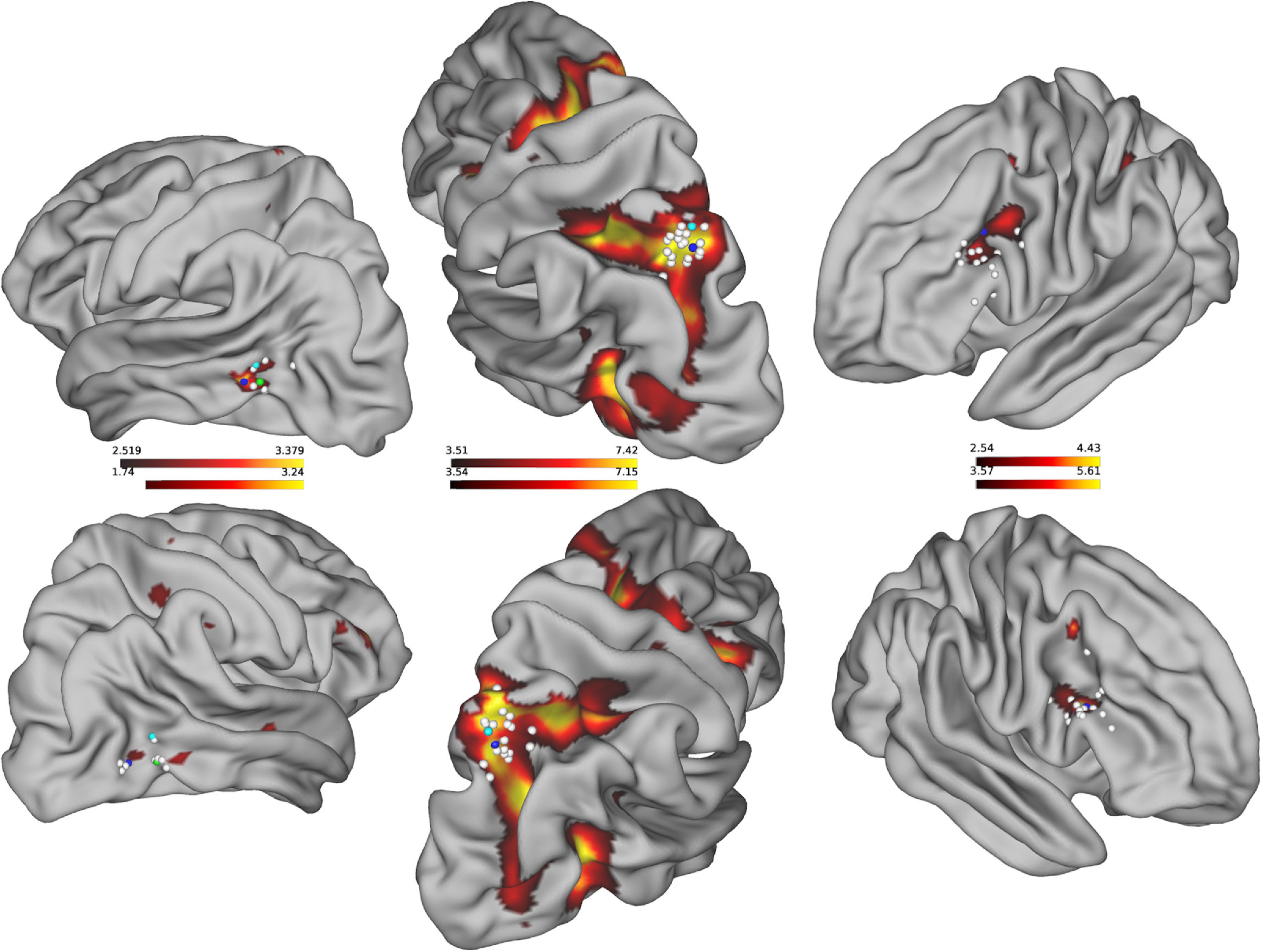
Activation maps emphasizing the ROIs. Left column, Contrast gaze following > color matching (localizer paradigm) used to identify the GFP. Blue dots mark maximum activation on the group level closest to locations taken from literature [green dots (Marquardt et al., 2017) and cyan dots (Materna et al., 2008)], white dots mark the maximum activation of those locations which were identifiable on the individual level. Middle column, Contrast gaze following > baseline fixation (localizer paradigm) used to identify saccade-related activity in the hLIP closest to location taken from (cyan dot; Sereno et al., 2001). Blue and white dots mark again, group level and individual coordinates. Right column, ambiguous-uninformative > unambiguous (contextual gaze following paradigm). Blue and white dots mark the group level and individual locations of the maximum IFJ-activity. See Extended Data Figure 3-1 for tabular form of all activated regions of each contrast and Extended Data Figures 3-2, 3-3, 3-4 for the respective contrast maps.

To localize the GFP we contrasted gaze following with color matching trials in the first experiment. At the group level (*N *=* *19) this contrast (gaze following > color matching) yielded a patch of significantly larger activity for gaze following close to the pSTS in both hemispheres. The contrast maxima (Fig. 3, left column, blue spheres) were located at *x*, *y*, *z =* −57, −61, −1 in the left and at *x*, *y*, *z = *48, −67, −1 in the right hemisphere. These locations closely match those known from previous studies, visualized as green and cyan spheres for comparison (Materna et al., 2008; Marquardt et al., 2017). In addition to the GFP, the gaze following > color matching contrast was also significant in a few more regions, not consistently seen as activated in previous work using the same paradigm (see Extended Data Fig. 3-1 for a list of all activated regions).

10.1523/ENEURO.0437-19.2020.f3-1 Extended Data Figure 3-1 List of activated brain areas. Download Figure 3-1, DOC file.

10.1523/ENEURO.0437-19.2020.f3-2 Extended Data Figure 3-2 Contrast map gaze following > color matching (*p *<* *0.001, cluster size > 6 voxels). Download Figure 3-2, EPS file.

10.1523/ENEURO.0437-19.2020.f3-3 Extended Data Figure 3-3 Contrast map gaze following > baseline (*p *<* *0.001, cluster size > 6 voxels). Download Figure 3-3, EPS file.

**Figure 4.**
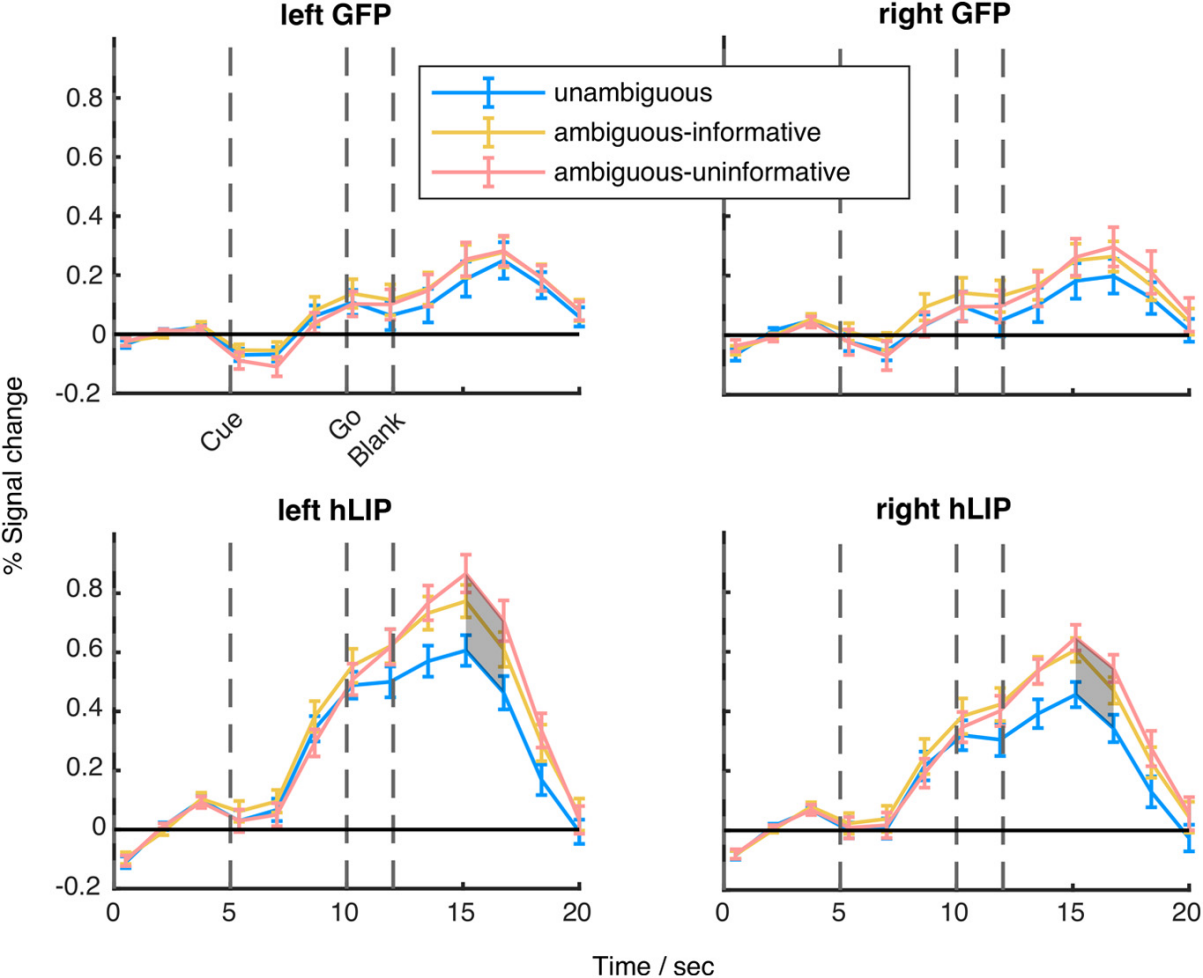
Time courses of activation in the GFP and the hLIP. Time course of mean percent signal change in the contextual gaze following experiment in areas identified in the localizer experiment (error bars are SEM). Areas in which conditions showed significant differences are shaded (permutations test, *q *<* *0.05). Vertical dashed lines represent the within trial events cue onset, go-signal, and blank fixation onset. See Figure 6 for the model-based analysis.

10.1523/ENEURO.0437-19.2020.f3-4 Extended Data Figure 3-4 Contrast map ambiguous-uninformative > unambiguous (*p *<* *0.001, cluster size > 6 voxels). Download Figure 3-4, EPS file.

We localized the right hemispheric GFP in nine individual subjects (mean distance to group coordinates = 6.6 mm, SD =* *3.1 mm) and the left GFP in six subjects (mean distance = 7.7 mm; SD =* *1.4 mm; Fig. 3, left column, white spheres).

An analogous procedure was applied to localize the hLIP, using the contrast gaze following versus baseline fixation. The location of maximum activation at the group level was found to be at *x*, *y*, *z = *21, −67, 50 (right; Fig. 3, middle column, blue spheres) and *x*, *y*, *z =* −21, −67, 53 (left; Fig. 3, middle column, blue spheres) in good accordance with previous work on saccade related activity in the parietal cortex (Sereno et al., 2001; Fig. 3, middle). We identified the hLIP regions bilaterally in all 19 subjects individually with a mean distance of 13.4 mm (SD =* *3.9 mm) to the standard coordinates in the right hemisphere and 11.93 mm (SD =* *3.7 mm) in the left hemisphere (Fig. 3, middle column, white spheres).

In order to determine whether BOLD activity in regions not delineated by the localizer experiment was modulated by the conditions of the contextual gaze following task, we contrasted activity in each of the ambiguous conditions with the unambiguous condition. The contrast ambiguous-uninformative > unambiguous was significant for a region in the inferior prefrontal cortex (Fig. 3, right) whose group level maxima were found in slightly different locations in the two hemispheres, namely at *x*, *y*, *z =* −39, 11, 29 in the left and *x*, *y*, *z = *48, 20, 23 in the right hemisphere (blue spheres), corresponding to the most lateral part of left BA 8 and upper right BA 44. In 15 subjects, we could delineate individual contrast locations [white spheres *ibidem*, SD of individual locations (in mm): right *x*, *y*, *z* = 5, 6, 6; left *x*, *y*, *z* = 5, 8, 6]. These individual locations were scattered around BA 44, BA 8 and BA 9 and henceforth we will refer to this region as the inferior frontal junction (IFJ). Contrasting ambiguous-informative > unambiguous yielded an overall weaker activation with only the right IFJ surviving a threshold of *p *<* *0.001.

Weaker, albeit still significant ambiguous-uninformative > unambiguous contrasts were also found in the medial part of left BA 8 at *x*, *y*, *z =* −3, 11, 50, bilaterally in BA 6 at *x*, *y*, *z =* −21, −4, 50 and *x*, *y*, *z = *24, −1, 50 and at *x*, *y*, *z = *36, 8, 47 (right hemisphere) not far from the IFJ (Extended Data Fig. 3-1). Upon reversing the contrast, i.e., unambiguous > ambiguous-informative/-uninformative, we observed bihemispheric significance within BA 13 (insula), BA 40, within the cingulate cortex (BA 24 and 31) and within BA 7 (all *p *<* *0.001, and a minimum of six adjacent voxel; Extended Data Fig. 3-1).

### Time course of BOLD signals

We wanted to know how the BOLD signal evolved over time, relative to the events of the trials in both of the a priori defined ROIs and the post hoc identified IFJ. Figure 4 shows the averaged time courses of the BOLD signal for each condition of the contextual gaze following experiment separately for the GFP and hLIP. This allows to present the response properties in the three conditions relative to baseline as well as their temporal pattern in relation to the cue and the go-signal not visible in the contrast-maps which only reflect the average signal of trials.

We performed two types of analyses to investigate the effects of context condition (unambiguous, ambiguous-informative and ambiguous-uninformative) on the BOLD activity: (1) permutation tests on each time point of the extracted BOLD signals (FDR corrected), (2) estimation of hierarchical models to infer BCIs of the time courses (Fig. 6).

In the GFP, we observed two peaks throughout the trial, one at 10 s and the other one after 16.5 s. Considering the latency of the BOLD signal of ∼5 s we assume that the first peak is related to the onset of the cue (at 5 s) and the second to the go-signal at 10 s. For the GFP, we did not observe significant difference between any conditions at any time point.

The hLIP region depicted a similar two-peak pattern in response to the cue and the go-signal. Statistical analysis indicated that the BOLD response was significantly different between unambiguous and ambiguous-uninformative trials after 15 s (permutation test, *q *<* *0.05; Fig. 4, bottom row) or after 13 s (BCI analysis; Fig. 6). The results of both analyses are in good agreement and, within the limits of the temporal resolution of the BOLD signal, suggest that the relevant event which caused the differentiation of the BOLD signal is the go-signal 10 s after trial onset rather than the cue 5 s after trial onset. Mind, that here, the model-based analysis method allowed a rejection of the null-hypothesis of no difference only for the left hemisphere yet not for the right one. Since the pattern of the right hemisphere closely resembles the one of the left hemisphere, and BCIs are only barely overlapping, we tend, however, to attribute this outcome to the low signal-to-noise ratio. This view is also supported by the decoding analysis (see below). There was no significant difference between the ambiguous-informative and ambiguous-uninformative conditions (*q *>* *0.05).

To rule out the possibility that the difference between the unambiguous and the ambiguous-uninformative condition was because of a larger number of saccades caused by the higher uncertainty, we performed a *t* test on the number of saccades across subjects, which yielded no significant difference (*p *>* *0.05). To summarize, hLIP exhibited a significantly stronger activity in ambiguous-uninformative trials compared with unambiguous trials (at least in the left hemisphere) while this was not the case for the GFP.

**Figure 5.**
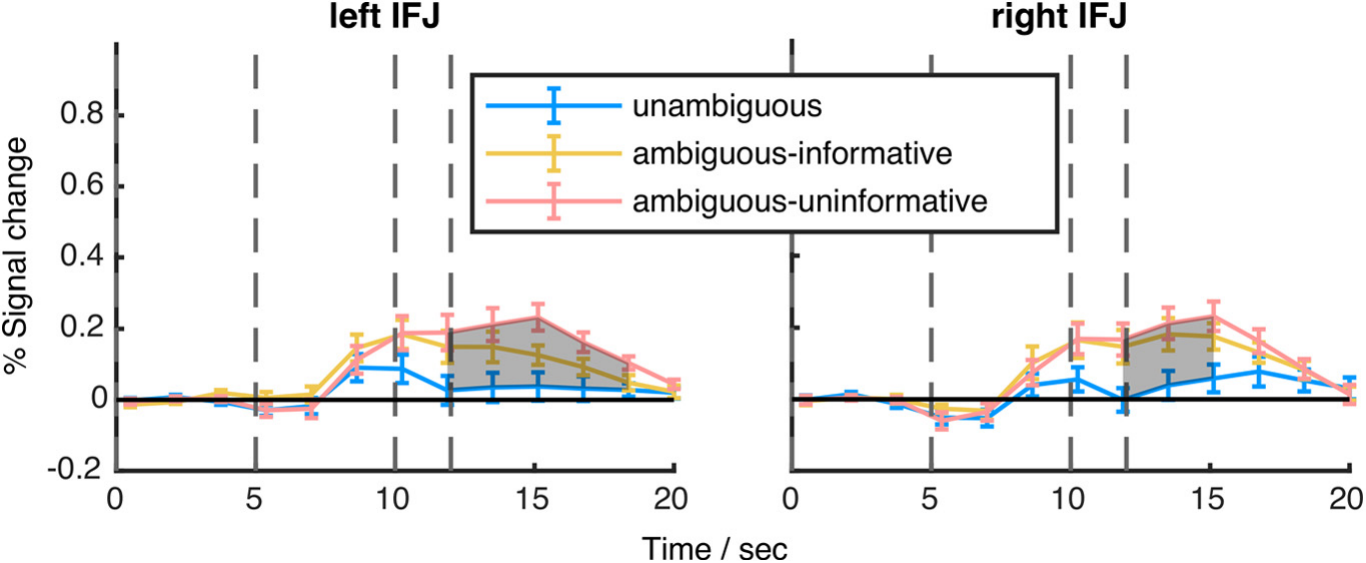
Time courses of activation in the IFJ. Time course of mean percent signal change during the contextual gaze following experiment of the IFJ (error bars are SEM). Areas in which conditions showed significant differences are shaded (permutations test, *q *<* *0.05). Vertical dashed lines represent the within trial events cue onset, go-signal, and blank fixation onset. See Figure 6 for the model-based analysis.

**Figure 6.**
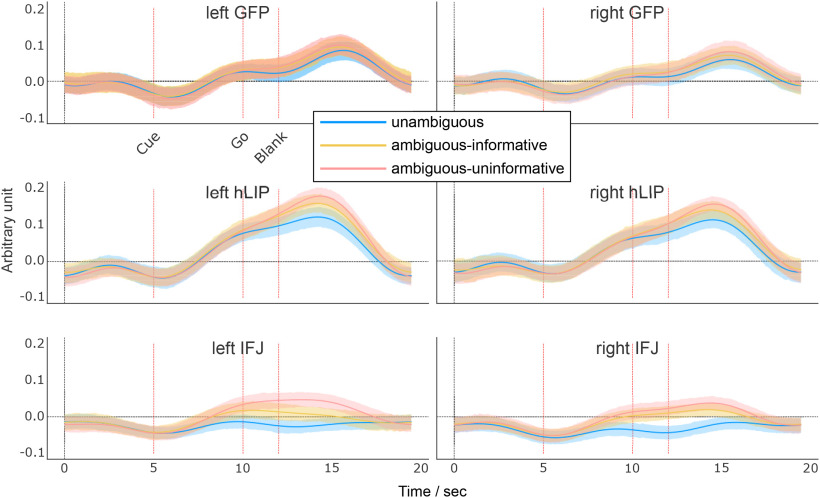
Model-based analyses of time courses during the contextual gaze following experiment. Lines depict the posterior means of BOLD activity. Shaded areas comprise 95% Bayesian credible intervals. Vertical dashed lines represent the within trial events cue onset, go-signal, and blank fixation onset.

Finally, we analyzed the time courses of the ROIs, identified in the exploratory whole-brain analysis. Of these ROIs (Extended Data Fig. 3-1), only the IFJ survived the permutation test (Figs. 5, 6). Notice that this analysis is not intended to compare the temporal average of the BOLD signal among the experimental conditions (which would be partly redundant to the activation map), but to get an idea about the temporal response characteristics during trials of the three conditions relative to baseline. Compared with GFP and hLIP, the condition-dependent BOLD signal in the IFJ exhibited a qualitatively different property: While the signal from the other two ROIs was modulated by the task during trials of all experimental conditions, the IFJ-signal did not exceed baseline signal during the unambiguous condition and was only modulated in the conditions comprising ambiguity. During the latter the signal was sustained at a higher level until the end of the trial. The permutation test yielded significant differences between the unambiguous and the ambiguous-uninformative conditions between 12 and 17 s (left) and 12 and 15 s (right; permutation test, *q *<* *0.05; Fig. 5) or between 9.5 and 16 s (BCI analysis; Fig. 6). Even if considering the 12 s-estimate as the onset of the differential, condition dependent modulation of the BOLD signal, the onset is too early to be related to the go-signal. Its association with the preceding event (the gaze and verbal cue 5 s earlier) seems more plausible. The profiles for the ambiguous-informative and ambiguous-uninformative conditions were statistically not different from each other. Summarizing, this analysis yielded that the BOLD signal from the IFJ significantly differentiated between conditions 3–4 s earlier than the hLIP BOLD signal, independent of the analysis method. With a temporal difference of 5 s between the main events of the task we tend to attribute the modulation of the IFJ-ROI to the gaze/auditory cue and the modulation of the hLIP-ROI the go-signal.

### Decoding of unambiguous versus ambiguous-uninformative

**Figure 7.**
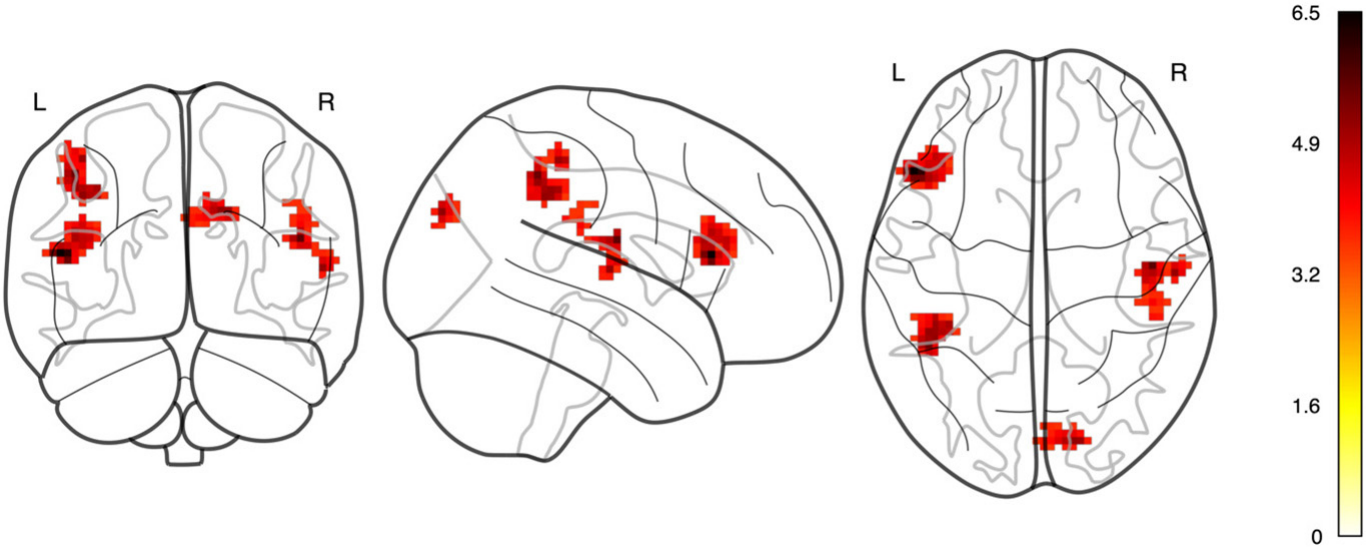
Group level result of the searchlight decoding analysis (t-map of classification accuracies, *p *<* *0.001). Extended Data Figure 7-1 shows the distributions of individual accuracies for the ROIs used in the time course analysis.

We also performed a decoding analysis by training a classifier on ambiguous-uninformative and ambiguous trials. The analysis yielded results which further support that only the parietal and the frontal regions but not the GFP are modulated by the contextual condition. The group-level t-map of classification accuracies (Fig. 7) evince hotspots in accordance with previously described locations. One qualification is that the hotspots in the parietal cortex are slightly lateral to the original hLIP-ROIs whose locations were estimated based on the study by Sereno et al. (2001). An additional area in BA 19 that was not visible in the contrast map provided a decodable signal as well. In accordance with the time course analysis, no temporal area contributed to the decoding of conditions.

10.1523/ENEURO.0437-19.2020.f7-1 Extended Data Figure 7-1 Classification accuracy distributions across participants for the ROIs used in the time course analysis. Asterisks marking distributions significantly different from a classifier performing at chance level (Wilcoxon signed-rank test, *p *<* *0.001). Download Figure 7-1, EPS file.

As a final step we extracted the mean accuracy values for voxels constituting the ROIs that were used for the time course analysis from each participant’s accuracy map and compared the obtained distributions against a classifier performing at chance level. Here, the only distributions that were significantly different from chance-level were those corresponding to the left and right IFJ-ROIs (Wilcoxon signed-rank test, *p *<* *0.001; both hemispheres). Neither the distributions stemming from the GFP-ROIs nor those from the original hLIP-ROIs were significantly different from chance level. Since we had found significant differences between unambiguous and ambiguous-uninformative conditions in the time course analysis of hLIP this is surprising at the first glance. We think that the explanation lies in the procedure we used to determine the individual hLIP locations. This procedure involved using coordinates of a different study as seed coordinates and thus may have biased the localization of the hLIP (Materials and Methods). Indeed, the contrast shown in Figure 3, middle column, indicates that the activity within the parietal cortex is widespread and spans the superior parietal lobule and the adjoining sulci. Consequentially, postulating a confined hLIP in the context of the β-contrasts may not be fully justified. However, the hotspot revealed by the decoding analysis may provide a more reliable estimate of the parietal area that is of relevance in our task and which is shifted in fronto-lateral directions as compared with the locations stemming from the β-contrasts.

Irrespective of this qualification regarding the anatomic delineation of hLIP, both the time course analysis and the decoding analysis suggest differentially activated regions in the parietal and frontal cortex while the GFP does not come into play. Thus, we see our main finding to be confirmed by two independent analyses.

## Discussion

In this study, we aimed at delineating the cortical regions that allow humans to single out objects being jointly attended to in case more than one object is hit by the other’s gaze vector. To this end we ran two experiments. In experiment 1 we identified two distinct cortical regions (GFP and hLIP) known from previous works to be involved in processing the other’s gaze direction and in shifting spatial attention respectively (Materna et al., 2008; Sereno et al., 2001; Marquardt et al., 2017). In experiment 2, subjects performed a contextual gaze following task in which they integrated gaze direction and auditory information to identify the objects attracting an avatar’s attention. While BOLD activity of the GFP was not modulated by the informativeness of the auditory information, hLIP showed increased activity when the provided information was insufficient to specify the target. The BOLD contrast between the condition unambiguously specifying the targets and the two ambiguous conditions identified yet another area only involved in contextual gaze following, missed by the localizer paradigm because of the lack of the need to disambiguate object choices. This area exhibited a continuously elevated response if and only if the evidence about the target was low. Unlike the other two areas, IFJ did not show a general response to events of the trials in all experimental conditions; apart from an initial bump resembling the early part of the activity profiles during the two ambiguous conditions, its activity during unambiguous trials was close to or undistinguishable from baseline activity. This pattern of the IFJ activation suggests that the area is involved in the process of selecting the target if sensory stimuli would not unequivocally single out the target, i.e., IFJ may provide a top-down attention/ selection signal.

This study confirms previous findings that the GFP, which is located close to the pSTS, plays a major role in processing information on the others’ gaze. Moreover, the present work shows that no matter if one or more potential target objects are hit by the other’s gaze vector, the BOLD activity in the GFP remains the same. The need to differentiate between objects in case more than one lies on the gaze vector requires contributions from additional areas that exhibit differential activity. One of these areas, the hLIP in the posterior parietal lobe is also activated in the traditional, restricted gaze following paradigms in which the gaze hits one object only. It is established that the hLIP is necessary for the control of spatial attention (Corbetta and Shulman, 2002). A role of hLIP is also supported by an additional decoding analysis that delineated voxels based on which the unambiguous and the ambiguous-uninformative conditions could be differentiated. This searchlight analysis revealed four hotspots, two of them in the posterior parietal cortex of both hemispheres, one in the left inferior prefrontal cortex and one in BA 19 of both hemispheres. The hotspots in the posterior parietal cortex turned out to be located slightly more rostral and inferior than the a priori defined hLIP regions as identified based on locations taken from Sereno et al. (2001). We do not think that this spatial incongruence is surprising since the two localization approaches were based on different tasks (hLIP from experiment 1 and the hotspots from experiment 2) and, moreover, relied on different analysis methods (β-contrasts vs decoding analysis). Hence, these two cortical spots, although overlapping or at least closely adjacent may not necessarily play the same role in gaze following. In any case, our statistical analyses showed that both, hLIP and the hotspots were sensitive to the experimental conditions. Future work may investigate the exact roles of both regions regarding contextual gaze following. The searchlight analysis also revealed a significant contribution of an area in BA 19, not identified by the original search for significant BOLD contrasts. BOLD activity in extrastriate BA 19 is typically seen in tasks that involve varying contributions to complex visual processing. Its functional role has remained elusive. In view of the fact that BA 19 of nonhuman primates maintains bidirectional connections with BA 7 (Rockland and Pandya, 1979) one may speculate that a significant contribution to the decoding of conditions decoding may arise from feedback connections originating in hLIP.

Work on monkey area LIP, arguably homologous to hLIP, has suggested that this area constitutes a priority or saliency map providing a representation of the environment that highlights locations that serve as attractors of attention. The saliency map may be modulated by bottom-up sensory cues, symbolic cues or gaze cues (Walther and Koch, 2006; Bisley and Goldberg, 2010). The latter is suggested by single unit recordings from area LIP. Many LIP neurons respond to the appearance of a gaze cue provided the gazed at location lies within the neuron’s receptive field (Shepherd et al., 2009). Spatial selectivity for gazed at locations and objects at these locations is also exhibited by many neurons in the monkey GFP (Ramezanpour and Thier, 2020). This selectivity suggests that the priority map in LIP might draw on input from the GFP. The yoked activation of hLIP/LIP and the GFP in BOLD imaging studies of gaze following is in principle in accordance with this scenario (Materna et al., 2008; Shepherd et al., 2009; Marquardt et al., 2017). However, the poor temporal resolution of the BOLD signals does not allow us to critically test whether the assumed direction of information flow holds true. In any case, bidirectional projections are known to connect monkey area LIP and parts of the STS (Seltzer and Pandya, 1994). One well-established pathway links area LIP and PITd, an area in the lower STS, probably close to the GFP, known to contribute to the maintenance of sustained attention (Stemmann and Freiwald, 2016; Sani et al., 2019). Yet, the anatomic data available does not allow us to decide whether the GFP does indeed contribute to this fiber bundle.

In the present study, the BOLD signal evoked by gaze following in hLIP was overall much stronger than in the GFP. Moreover, unlike the GFP signal, it exhibited a dependence on the conditions of the contextual gaze following experiment. Higher activity was associated with the ambiguous-informative and the ambiguous-uninformative conditions, both associated with unresolved uncertainty about the object requiring a decision of the participant that could only partially be based on information provided by the cue. Why should a region thought to coordinate spatial shifts of attention show an influence of target ambiguity, i.e., the need to choose between several potential targets? One possible answer may be that the higher hLIP activity reflects an increased attentional load. More specifically, increased uncertainty in ambiguous trials may have prompted more shifts of attention from one object to the other in an attempt to resolve the ambiguity. Although we found no difference in the number of exploratory saccades after the *go*-signal across conditions, we cannot rule out that participants covertly shifted attention between targets in ambiguous trials more than in the other trials. However, a more parsimonious explanation could be that hLIP constitutes a neural substrate for making decisions under uncertainty independent of the attentional load as suggested by several studies such as Vickery and Jiang (2009).

The BOLD signal in the area we identified as the IFJ (between premotor cortex (BA 6), BA 44 and BA 8) exhibited a dependency on condition as well. This result is suggested by the BOLD contrast (Fig. 3, right column), the time course analysis (Figs. 5, 6) as well as by the decoding analysis (Fig. 7; Extended Data Fig. 7-1). However, the time course analysis revealed a fundamental difference compared with response profiles of BOLD activity in hLIP or the GFP. Sustained activity could only be observed in trials of the two ambiguous conditions, i.e., when the participants needed to make decisions under sensory uncertainty. This suggests that the condition dependency of the IFJ signal may be a consequence of shifts of attention between the two object categories, houses and hands. This interpretation draws on a MEG-fMRI study that demanded the allocation of attention to distinct classes of visual objects such as faces and spatial scenes (Baldauf and Desimone, 2014). Depending on the object of attention, γ band activity in the IFJ was synchronized either with the fusiform face area (FFA) or the parahippocampal place area (PPA). Additional support for this view comes from spatial cueing paradigms, which suggest that the IFJ primarily supports transient attentional processes, such as covert attentional shifts (Asplund et al., 2010; Tamber-Rosenau et al., 2018). We speculate that the time course of activity in the IFJ reflects the coordination of covert shifts of attention until the choice for the saccade target is made. In unambiguous trials, the lack of ambiguity allows fast decisions and since no attentional shifts are necessary the IFJ is not required.

The functional characteristics of the GFP, hLIP and the IFJ attribute complementary functions to each area which, in sum, allows gaze following under sensory ambiguity. We propose that information on the direction of the other’s gaze is provided by the GFP and modulates the saliency map generated by area hLIP such that spatial positions in the direction of the gaze vector are highlighted. In this situation the choice of which of the possible objects is the most relevant one requires the resolution of uncertainty which is accomplished by the IFJ. In this scenario the intersection between the spatial information provided by the GFP-hLIP complex and the object-based information provided by the IFJ singles out one object that will then become the target of the observer’s gaze following response, elicited by the hLIP.

Several points need to be addressed by future work to test and to further refine this concept. As a first step, it will be necessary to investigate the temporal interplay between these regions in an attempt to establish causal interactions to critically test the model. Our hypothesis assumes that the IFJ has a leading role in processing information on competing objects on the gaze vector, resolving the uncertainty as to which one the target is. The conclusion that IFJ has a leading role in the disambiguation of the object set is primarily based on the fact that ambiguity related information arises first in IFJ and only later in hLIP. Yet, we cannot rule out that this sequence might be an artifact of region-specific differences in the statistical power of the BOLD time course analysis, eventually in conjunction with region specific differences in the variability of BOLD signal latencies.

To summarize, our results suggest a fronto-temporo-parietal network for geometric gaze following and the allocation of joint attention. While the GFP seems to have a leading role in selecting objects identified by the other’s gaze vector, the IFJ seems to play a central role in disambiguating object choices in case more than one object may be hit by the vector. Finally, the hLIP acts as a priority map, highlighting the spatial location of the target object based on the focus of attention, and contribute to the execution of gaze shifts.

## Synthesis

Reviewing Editor: Tatyana Sharpee, The Salk Institute for Biological Studies Decisions are customarily a result of the Reviewing Editor and the peer reviewers coming together and discussing their recommendations until a consensus is reached. When revisions are invited, a fact-based synthesis statement explaining their decision and outlining what is needed to prepare a revision will be listed below. The following reviewer(s) agreed to reveal their identity: Winrich Freiwald, Stephen Shepherd.

The manuscript “Frontal, parietal and temporal brain areas are differentially activated when disambiguating potential objects of joint attention", Kraemer et al., describes interesting and relevant piece of work on the neural mechanisms of gaze following. Please address concerns raised by the reviewers, paying particular attention to the concerns about statistical analyses.

Reviewer 1

Statistics 1: Results on IFJ constitute, partly, double dipping. The area was defined for the same (or a highly correlated) contrast to the one whose significance is then re-evaluated. I would ask the authors to state how they are addressing this concern, as I fail to see how it has been addressed.

Statistics 2: We cannot be very sure about similarity of GFP activations in the three ambiguity conditions. The response looks, qualitatively, just like the one in hLIP and IFJ - just that it does not reach significance. We do not know how strongly the different time points are correlated with each other, and thus it is unclear how to think about these differences. But in any case, it is a negative result, an absence of evidence for a functional differentiation (by some significance test). One could also look at this differently, e.g. look at decodability of the three conditions, voxel by voxel, or one could look at whether across the three areas the conditions differentiate. In any case, the results as they stand caution against a strong interpretation of functional segregation of the three areas. Again, I would ask the authors to state how they are addressing this concern.

I believe that the time-course analysis is not very informative. It mainly serves to inform the aforementioned points on significance and on overall activation, but no serious attempt is made to use it for attributing activations to different components of the task. The fact that significance measures differ in timing between hLIP and IFJ, given the very different degrees of activation the authors are highlighting, does not really carry much meaning.

Figures and Tables. Figure 3 should show both positive and negative contrasts. To facilitate comparison, the brains should be oriented in the same way for the three contrasts (who would better be shown in the figure in addition to the legend), and significant modulations from contrasts 1 and 3 (left and right) shown as outlines in the middle column. The authors may, of course, choose a different way to facilitate comparison between the three comparisons. It would be important to know whether contrast 2 encompasses GFP and IFJ as well. Figures 4 and 5 should be fused and show the ROI used in each row. Please make sure that all acronyms are defined and that the same nomenclature is used (e.g. Fig. 1 and current 5)! Fig. 2: use of acronyms au etc. in figure not necessary and should be replaced with full description. Axis labels are missing.

Reviewer 2:

It seemed to me that the manuscript should clarify that while it does provide evidence supporting previous reports of a pSTS GFP and of hLIP, the statistics aren’t strong enough to serve as independent verification of these findings. However, I agree the reported results *are* strong enough to localize these previously-established cortical patches and subject them to supplemental analysis, specifically, of their time-series activity in relationship to cognitive processes. For these narrower headline results, appropriate statistics (FDR, BCI, permutations) appear to have been conducted.

Some details were unclear to me: for example, I didn’t understand how BCI are said to differ from CI in lines 300-2.

Also, I didn’t see a legend for the statistics table, but it seemed like the “power” column was speaking to statistical significance testing rather than to “power” per se? Perhaps I misunderstood.

I’m concerned that the English could use another pass. There are multiple instances of subtle issues which could cloud understanding: For example, in line 5, you mean to say hLIP mediates a process (“mediated by”), not that it provides evidence for a claim (“based on”).

Also, it seems like there might be some relevant work which has been skipped over. For example, isn’t there prior evidence that even unambiguous gaze following integrates reflexive and social-contextual information? Moreover, I think there might be some prior data on frontal-lobe contributions to gaze following which are worth review. I don’t have access to my library at the moment, but I wonder if your IFJ ROI overlaps the IFG locus in Newman-Norlund..Bekkering 2010 “Virtual lesions of the IFG abolish response facilitation for biological and non-biological cues.”
