# Supplementary material for: Frontal, Parietal, and Temporal Brain Areas Are Differentially Activated When Disambiguating Potential Objects of Joint Attention
Source: eNeuro. 2020 Oct 19;7(5):ENEURO.0437-19.2020. doi: 10.1523/ENEURO.0437-19.2020 (PMC7581189; doi:10.1523/ENEURO.0437-19.2020)
Supplement: Extended Data Figure 2-1 — Description of the localizer paradigm. Download Figure 2-1, DOC file. [file enu-eN-NWR-0437-19-s02.doc]

*Localizer experiment*

As a localizer task we used a cued saccade task, also denoted as a *gaze following vs. color mapping* task (Marquardt et al., 2017). During a baseline fixation phase, subjects had to fixate on a red dot between the eyes of a photography of a face gazing straight ahead. Below the stimulus face, five colored and horizontally arranged rectangles were presented as gaze targets. After five seconds of baseline fixation, the portrait´s eye-gaze shifted towards one of the targets and, simultaneously, its eye color (i.e. the color of the irises) changed to match the color of one of the rectangles. After one second, the red dot disappeared (go signal) and the subjects had to shift their own gaze towards to the correct target and fixate it. There were two different experimental conditions: (1) in *gaze following* trials, the correct target was determined by the eye-gaze direction of the stimulus face, (2) in *color mapping* trials, the correct target had the same color as the stimulus irises. The task was performed in several runs, each consisting of four blocks (2 gaze following, 2 color mapping). Each block started with the task instruction as a seven seconds lasting window containing the written words “gaze following” or “color mapping”, followed by 10 corresponding trials. Task instruction alternated between blocks. Target objects were counter-balanced such that each rectangle was the target object twice during a block and target order was pseudorandomized.
