# Supplementary material for: Frontal, Parietal, and Temporal Brain Areas Are Differentially Activated When Disambiguating Potential Objects of Joint Attention
Source: eNeuro. 2020 Oct 19;7(5):ENEURO.0437-19.2020. doi: 10.1523/ENEURO.0437-19.2020 (PMC7581189; doi:10.1523/ENEURO.0437-19.2020)
Supplement: Extended Data Figure 3-1 — List of activated brain areas. Download Figure 3-1, DOC file. [file enu-eN-NWR-0437-19-s03.doc]

**Figure 3-1**:

| **Corresponding Area** | **Contrast** | **x** | **y** | **z** | **Threshold** |
| --- | --- | --- | --- | --- | --- |
| Left-Fusiform (GFP) | *gaze-following*  >  *color-matching* | -57 | -61 | -1 | 0.01, 6 Voxel |
| Right-Fusiform (GFP) |  | 48 | -67 | -1 |  |
| Outside defined BAs (Colliculus) |  | -6 | -34 | -16 |  |
| Outside defined BAs (Colliculus) |  | 9 | -34 | -16 |  |
| Right BA45 |  | 45 | 32 | 8 |  |
| Left BA8 (IFJ) | *ambiguous-uninformative*  > *unambiguous* | -39 | 11 | 29 | 0.001, 6 Voxel |
| Right BA44 (IFJ) |  | 48 | 20 | 23 |  |
| Medial BA8 |  | -3 | 11 | 50 |  |
| Left BA6 |  | -21 | -4 | 50 |  |
| Right BA6 |  | 24 | -1 | 50 |  |
| Outside defined BAs |  | 36 | 8 | 47 |  |
| Left Insula | *unambiguous*  >  *ambiguous-uninformative* | -36 | -16 | 5 | 0.001, 6 Voxel |
| Right Insula |  | 42 | -19 | -1 |  |
| Left BA40 |  | -63 | -28 | 20 |  |
| Right BA40 |  | 51 | -31 | 17 |  |
| Left BA24 (Cingulate cortex) |  | -9 | -34 | 44 |  |
| Right BA 31 (Cingulate cortex) |  | 9 | -16 | 41 |  |
| BA7 |  | -24 | -43 | 65 |  |
| BA7 |  | 12 | -46 | 65 |  |
| Right BA44 (IFJ) | *Ambiguous-informative*  > *unambiguous* | 45 | 11 | 26 | 0.001, 6 Voxel |
